# Role of inducible nitric oxide synthase pathway on methotrexate-induced intestinal mucositis in rodents

**DOI:** 10.1186/1471-230X-11-90

**Published:** 2011-08-16

**Authors:** Renata FC Leitão, Gerly AC Brito, Reinaldo B Oriá, Manuel B Braga-Neto, Emmanuelle AL Bellaguarda, Johann V Silva, Antoniella S Gomes, Roberto CP Lima-Júnior, Francisco JWS Siqueira, Rosemeyre S Freire, Mariana L Vale, Ronaldo A Ribeiro

**Affiliations:** 1 Department of Physiology and Pharmacology, Federal University of Ceara, Fortaleza, Brazil; 2 Department of Morphology, Federal University of Ceará, Fortaleza, Brazil

**Keywords:** Nitric oxide, Nitric oxide synthase, Methotrexate, Aminoguanidine, Nφ-Nitro-L-arginine methyl ester

## Abstract

**Background:**

Methotrexate treatment has been associated to intestinal epithelial damage. Studies have suggested an important role of nitric oxide in such injury. The aim of this study was to investigate the role of nitric oxide (NO), specifically iNOS on the pathogenesis of methotrexate (MTX)-induced intestinal mucositis.

**Methods:**

Intestinal mucositis was carried out by three subcutaneous MTX injections (2.5 mg/kg) in Wistar rats and in inducible nitric oxide synthase knock-out (iNOS^-/-^) and wild-type (iNOS^+/+^) mice. Rats were treated intraperitoneally with the NOS inhibitors aminoguanidine (AG; 10 mg/Kg) or L-NAME (20 mg/Kg), one hour before MTX injection and daily until sacrifice, on the fifth day. The jejunum was harvested to investigate the expression of Ki67, iNOS and nitrotyrosine by immunohistochemistry and cell death by TUNEL. The neutrophil activity by myeloperoxidase (MPO) assay was performed in the three small intestine segments.

**Results:**

AG and L-NAME significantly reduced villus and crypt damages, inflammatory alterations, cell death, MPO activity, and nitrotyrosine immunostaining due to MTX challenge. The treatment with AG, but not L-NAME, prevented the inhibitory effect of MTX on cell proliferation. MTX induced increased expression of iNOS detected by immunohistochemistry. MTX did not cause significant inflammation in the iNOS^-/- ^mice.

**Conclusion:**

These results suggest an important role of NO, via activation of iNOS, in the pathogenesis of intestinal mucositis.

## 1. Background

Mucositis is a debilitating side effect of cytotoxic chemotherapy and radiotherapy. It involves inflammation and mucosal ulceration of the alimentary tract, resulting in symptoms including pain, abdominal bloating, nausea, vomiting and diarrhea, and may significantly impair treatment compliance [1,2].

It has been demonstrated that methotrexate (MTX), an inhibitor of dihydrofolate reductase and of DNA synthesis, can disrupt the intestinal epithelial barrier [3], leading to mitotic arrest in the crypts and villous blunting [4,5]. The main mechanism behind the development of mucositis was considered to be the result of direct cytotoxic effects of chemotherapy or radiotherapy on the basal cells of the epithelium because of its high cell turnover rate. Subsequently, researchers investigating intestinal damage, found that, following radiation, the primary damage response occurred in endothelial cells [6,7]. It is postulated that mucositis occurs in five overlapping phases: initiation, up-regulation and message generations, signaling and amplification, ulceration and healing. [2,8].

Cytokines have been shown to stimulate the expression of the inducible NOS synthase isoform (iNOS) with consequent production of nitric oxide (NO). Nitric oxide (NO) is a free radical associated with a multitude of physiological functions. This highly reactive molecule is synthesized from L-arginine by a group of isoenzymes collectively termed NO synthases (NOS). NOS exists as three distinct isoforms, the constitutive endothelial (eNOS) and neuronal (nNOS) NOS isoforms, and the inducible NOS variant (iNOS). [9-12]. The physiological role of NO can be examined by blocking NOS using some efficient inhibitors such as Nφ-Nitro-L-arginine methyl ester (L-NAME) and aminoguanidine. L-NAME is a competitive and non-selective inhibitor of NOS [13]. Aminoguanidine inhibits particularly the inducible NOS isoform [14]. Our group has previously demonstrated the participation of NO, by usage of those NOS inhibitors, in the pathogenesis of oral mucositis induced by 5-fluorouracil [15].

Although NO is important in host defense and homeostasis, it is also regarded as harmful and has been implicated in the pathogenesis of a wide variety of inflammatory and autoimmune diseases [10]. NO exerts its effects directly or via the formation of potent oxidants [16]. During inflammatory reactions, large amounts of NO and superoxide are formed and may lead to the peroxynitrite anion, a toxic product of NO combined with superoxide, which can nitrate the phenolic ring of tyrosine residues in proteins [17]. Accordingly, a recent study by Kolli et al demonstrated that nitrosative stress may play a role in MTX-induced intestinal damage. Following treatment with MTX, they found increased staining of nitrotyrosine and of nitrate levels in the intestinal samples, which was accompanied by neutrophil infiltration [18]. However, the specific role of the inducible form of NOS and the effect of NOS inhibitors was not evaluated.

Thus, the aim of this study was to investigate the effect of nitric oxide (NO) on the pathogenesis of methotrexate-induced intestinal mucositis, looking at specifically the role of the inducible form of iNOS and the effect of NOS inhibitors.

## 2. Methods

### 2.1. Animals

Forty-eight male Wistar rats, weighing 140 to 160 g, were obtained from the Federal University of Ceará and eight C57BL/6 inducible nitric oxide synthase knock-out mice (iNOS^-/- ^) and corresponding wild-type animals (iNOS^+/+^), weighing 22 to 25 g, were obtained from the Animal Facility located at the Faculty of Medicine of Ribeirão Preto, University of São Paulo. All animals were housed in temperature-controlled rooms and received water and food ad libitum. Surgical procedures and animal treatments were conducted in accordance with the Institutional Animal Care and Use Committee guidelines from both Universities.

### 2.2. Materials

Nφ-Nitro-L-Arginine Methyl Ester (L-NAME), aminoguanidine and L-arginine were purchased from Sigma-Aldrich (St. Louis, MO, U.S.A.). The methotrexate (MTX) used in this study is a product of Faulding (Maine, Australia). Rabbit anti-NOS-2 and Biotinylated goat anti-rabbit were purchased from Santa Cruz Biotechnology (Santa Cruz, CA, U.S.A). Rabbit anti-nitrotyrosine was purchased from Upstate^® ^(Lake Placid, NY, U.S.A). Mouse anti-rat Ki67 and biotinylated rabbit anti-mouse were purchased from DakoCytomation. Vectastatin^® ^ABC detection system and the VIP substrate kit used in immunohistochemistry were obtained from Vector Laboratories (Burlingame, CA, U.S.A.). The apoptosis assay was performed using the ApopTag Peroxidase In Situ Detection Kit (Chemicon International, USA). Proteinase K was from Sigma-Aldrich (St. Louis, MO, U.S.A.).

### 2.3. Induction of Experimental Intestinal Mucositis

Intestinal mucositis was induced by three subcutaneous (s.c.) MTX administrations on the first three days of the experiment (2.5 mg/kg), according to a model previously described [19] and modified by our laboratory. The animals were sacrificed on the 5^th ^day after the first injection of MTX, under deep anesthesia with chloral hydrate (250 mg/kg, i.p.).

### 2.4. Experimental design

The Wistar rats groups with intestinal mucositis were treated intraperitoneally (i.p.) with either the NOS inhibitors aminoguanidine (10 mg/Kg) or L-NAME (20 mg/Kg), one hour before the mucositis induction and daily until sacrifice (on the 5^th ^day). Control groups including animals not subjected to intestinal mucositis (control) and a group which was subjected to the experimental mucositis and received saline i.p. (MTX).

In another set of experiments, iNOS^-/- ^and wild-type mice (iNOS^+/+^) were divided into 4 groups of 4 animals each, as follow: knock-out animals submitted to intestinal mucositis (iNOS^-/-^/MTX), a group of wild-type mice which was also submitted to intestinal mucositis (iNOS^+/+^/MTX) and two control groups consisting of knock-out mice and corresponding wild-type animals not subjected to intestinal mucositis (iNOS^-/-^/control and iNOS^+/+^/control, respectively).

### 2.5. Histopathology Analysis

On day 5, after killing, the intestines (duodenum, jejunum and ileum) were dissected. In each experiment, samples were removed for histopathological analysis. The specimens were fixed in 10% (v/v) neutral-buffered formalin, dehydrated and embedded in paraffin. Sections were cut and stained with haematoxylin and eosin (H&E) and examined by light microscopy (Leica DM LS 2 - Wetzlar, Germany) by an experienced histologist blinded to the experimental groups and damage extent. The severity of mucositis was graded using a modification of the Macpherson and Pfeiffer histopathological grading system [20] previously described [21], considering microscopic findings such villus and crypt integrity, inflammatory cell influx, vacuolization and edema. (Table 1).

**Table 1:** Histopathological grading scores

Scores	Microscopic findings
**0**	Normal histological findings
**1**	*Mucosa: *villus blunting, loss of crypt architecture, sparse inflammatory cell infiltration, vacuolization and edema. Normal muscular layer
**2**	*Mucosa: *villus blunting with fattened and vacuolated cells, crypt necrosis, intense inflammatory cell infiltration, vacuolization and edema Normal muscular layer
**3**	*Mucosa: *villus blunting with fattened and vacuolated cells, crypt necrosis, intense inflammatory cell infiltration, vacuolization and edema *Muscular: *edema, vacuolization, sparse neutrophil infiltration

### 2.6. Intestinal Morphometry

Villus height was measured from hematoxylin and eosin slides on a light microscope equipped with a high-resolution digital camera Leica DFC 320 (Wetzlar, Germany), connected to a computer with an image captured program. Villus height was measured from the baseline to the villus tip. At least 10 clear longitudinal villi sections were selected and counted for each sample (six samples for each group). All morphometric measurements were done blindly with Leica Measure Software (Wetzlar, Germany).

### 2.7. Myeloperoxidase assay

Segments of duodenum, jejunum and ileum were stored at -70°C until required for the assay. After homogenization and centrifugation (4,500 G, 20 min), myeloperoxidase activity associated with neutrophil azurophilic granules was determined by a colorimetric method described previously [22]. Results were reported as MPO units/mg of tissue. The unit of MPO activity was defined as the one converting 1 μmol/min of hydrogen peroxide into water at 22°.

### 2.8. Cell proliferation and cell death

Methotrexate-induced cell death was investigated on the 5^th ^day using TdT-mediated dUTP nick end-labeling (TUNEL) method. Briefly, paraffin-embedded jejunal sections were rehydrated and incubated with 20 μg/mL of proteinase K for 15 minutes at room temperature. Endogenous peroxidase was blocked by treating with 3% (v/v) hydrogen peroxide in PBS for 5 minutes at room temperature. After washing, sections were incubated in a humidified chamber at 37°C for 1 h with TdT buffer containing TdT enzyme and reaction buffer. Specimens were incubated for 10 minutes at room temperature with a stop/wash buffer and then incubated in a humidified chamber for 30 minutes with anti-digoxigenin peroxidase conjugate at room temperature. After a series of PBS washes, slides were covered with peroxidase substrate to develop color and then washed in three changes of dH_2_O and counterstained in 0.5% (w/v) methyl green for 10 minutes at room temperature. The TUNEL positive cells were counted (10 fields per slide; x1000) in order to perform a statistical comparison.

Crypt cell proliferation was assessed on the 5^th ^day by Ki67 immunohistochemistry, a nuclear antigen that is present in proliferating cells but absence in quiescent cells [23,24]. Jejunum tissues from each experimental group were immunostained using the streptavidin-biotin-peroxidase method, as described elsewhere [25]. The Ki67 positive cells were counted (10 fields per slide; x1000) in order to perform a statistical comparison.

### 2.9. Immunohistochemistry for iNOS and nitrotyrosine

Nitrotyrosine and iNOS immunohistochemistry was performed using the streptavidin-biotin-peroxidase method [25]. Briefly, sections were deparaffinized and rehydrated through xylene and graded alcohols. After antigen retrieval, endogenous peroxidase was blocked (15 min) with 3% (v/v) hydrogen peroxide and washed in phosphate-buffered saline (PBS). Sections were incubated overnight (4°C) with either a primary rabbit anti-iNOS antibody diluted 1:200 or rabbit anti-nitrotyrosine antibody diluted 1:400 in PBS plus bovine serum albumin (PBS-BSA). Slides were then incubated with biotinylated goat anti-rabbit; diluted 1:200 in PBS-BSA. After washing, slides were incubated with avidin-biotin-horseradish peroxidase conjugate (Strep ABC complex by Vectastain^® ^ABC Reagent and peroxidase substrate solution) for 30 minutes, according to the Vectastain protocol. iNOS was visualized with the chromogen 3.3'diaminobenzidine (DAB). Negative control sections were processed simultaneously, as described above, but with the first antibody replaced by PBS-BSA 5%. None of the negative controls showed iNOS or nitrotyrosine immunoreactivity. Slides were counterstained with Harry's hematoxylin.

### 2.10. Statistical Analysis

Data were described as either means ± SEM or median, as appropriate. Analysis of Variance (ANOVA) followed by Bonferroni's test was used to compare means and Kruskal-Wallis and Dunns tests to compare medians; *P *< 0.05 was defined as statistically significant.

## 3. Results

### 3.1. Histopathology and Morphometry analysis

**Figure 1:**
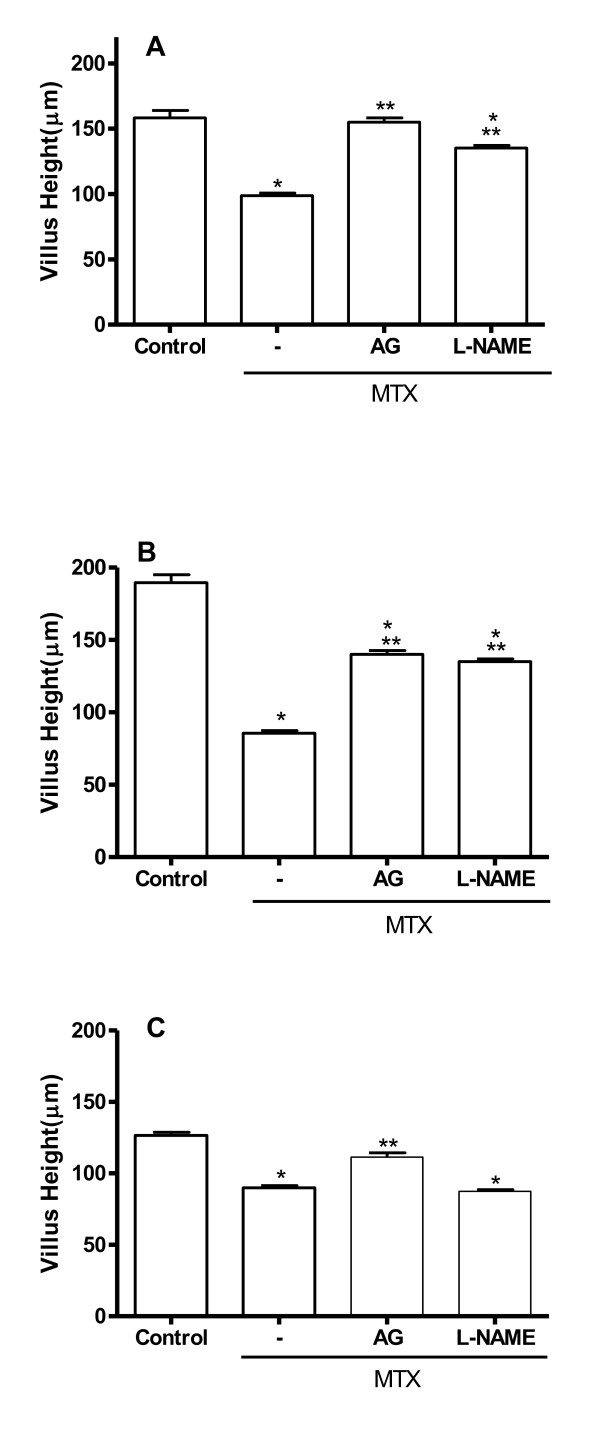
**Effect of aminoguanidine and L-NAME on MTX-induced villus atrophy in the duodenum (A), jejunum (B), and ileum (C)**. Bars represent the mean value ± standard error of the mean (SEM) of the villus height in each segment. *p < 0.05 represents statistical differences compared to control group. **p < 0.05 represents statistical differences compared to MTX group treated with saline (-). The number of animals in each group was at least six. Data were analyzed by using analysis of variance (ANOVA) and Bonferroni tests.

**Figure 2:**
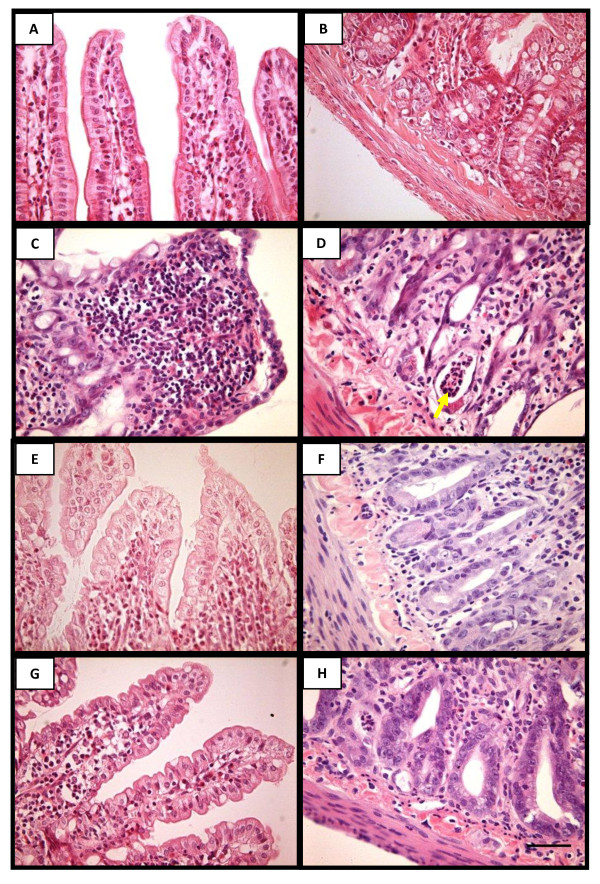
**Representative photomicrographies of normal jejunum showing the region of villi (**A**) and crypt (**B**); jejunum of MTX treated rat showing blunted villous recovered with flattened and vacuolated cells, and presenting inflammatory cell infiltration on the lamina propria (**C**) and crypt necrosis with neutrophil infiltration (**D**); systemic administration of aminoguanidine (**E **and **F**) and L-NAME (**G **and **H**) reduced the villus height atrophy as well as crypt destruction in the segments of jejunum**. Hematoxylin and eosin staining. Magnification 400x. Scale bar lengths 10 μm. The arrow indicates neutrophils within a necrotic crypt.

MTX administration induced a significant (p < 0.05) villus atrophy in all three small intestinal segments (duodenum, jejunum and ileum) when compared with the group not subjected to intestinal mucositis (control) (Figure 1). The villi from MTX-treated rats exhibited flattened and vacuolated cells. Additionally, the histopathology revealed crypt necrosis and inflammatory infiltration within the lamina propria, constituted by mononuclear and polimorphonuclear cells in the MTX group (Figure 2). Both aminoguanidine and L-NAME prevented the villus atrophy in the duodenum and jejunum, observed on day 5. Aminoguanidine, but not L-NAME, significantly prevented the villus atrophy in the ileum (Figure 1). Both aminoguanidine and L-NAME reduced villus blunting and crypt necrosis in the three intestinal segments as can be clearly observed in representative jejunal section seen in Figure 2.

The histopathology of the small bowel segments (duodenum, jejunum and ileum) of animals subjected to MTX-induced intestinal mucositis, on day 5, showed accentuated villus blunting, covered with flattened and vacuolated cells, crypt necrosis, intense inflammatory cell infiltration, and edema both in mucosa and muscular (MTX histopathological score 3; control histopathological score 0) (control-Figure 2A and 2B; MTX- Figure 2C and 2D; Table 2). The treatment with both aminoguanidine (Figure 2E and 2F; Table 2) and L-NAME (Figure 2G and 2H; Table 2) significantly (p < 0.05) reduced villus and crypt damages and inflammatory alterations in the all three small intestine segments when compared to MTX group (MTX histopathological score 3; AG and L-NAME histopathological score 1) (Figure 2A and 2B; Table 2).

**Table 2:** Effect of aminoguanidina (AG) and L-NAME on microscopic findings of duodenum, jejunum and ileum of animals submitted to methotrexate (MTX)-induced intestinal mucositis, observed on 5^th ^day

	Duodenum	Jejunum	Ileum
Control	0 (0-0)	0 (0-0)	0 (0-0)
MTX	3 (3-3)*	3 (3-3)*	3 (3-3)*
AG	1 (1-1)*, **	1 (1-1)*, **	1 (1-1)*, **
L-NAME	1(1-2)*, **	1 (1-1)*, **	1 (1-1)*, **

Intestinal mucositis was carried out by three subcutaneous MTX injections (2.5 mg/kg) in Wistar rats. Rats were treated intraperitoneally with the NOS inhibitors aminoguanidine (AG; 10 mg/Kg) or L-NAME (20 mg/Kg), one hour before MTX injection and daily until sacrifice, on the fifth day. Data represents the median values (and range) of microscopic scores in at least 10 animals per group. *p < 0.05 compared to normal animals (control group); **p < 0.05 compared to animals submitted to MTX-induced intestinal mucositis that received saline, observed on the 5^th ^day. Data were analysed by using Kruskal-Wallis and Dunn's test.

### 3.2. Mieloperoxidase activity

**Figure 3:**
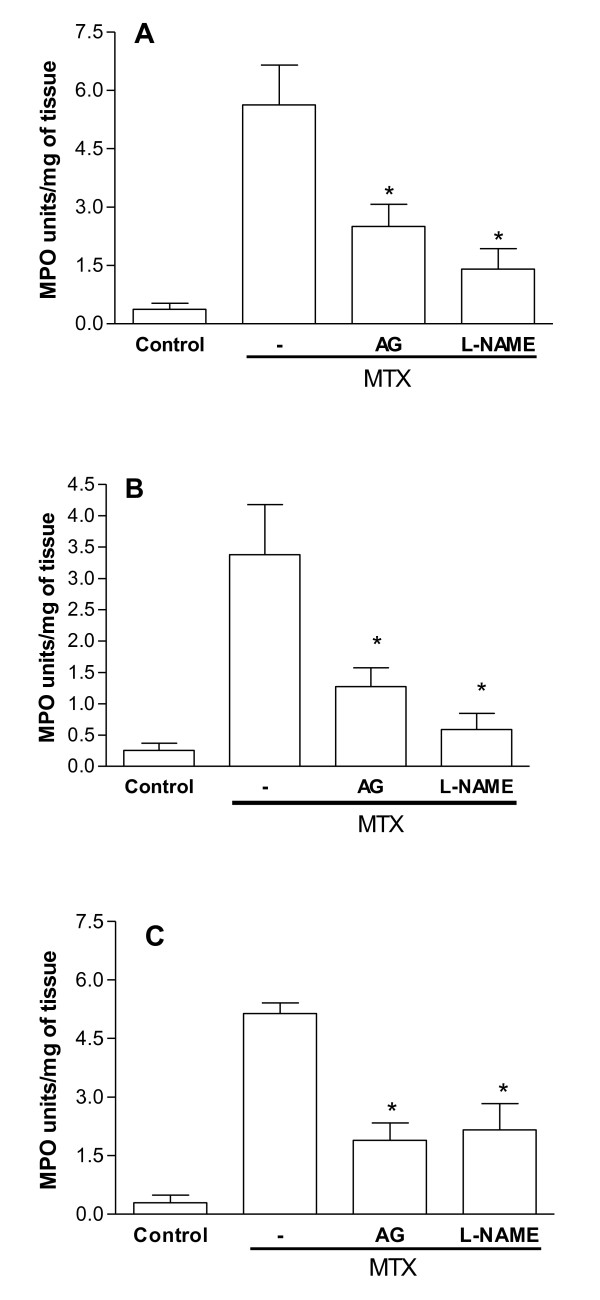
**Effect of aminoguanidine and L-NAME on myeloperoxidase (MPO) activity in the duodenum, jejunum and ileum segments of rats submitted to intestinal mucositis**. Bars represent the mean value ± standard error of the mean (SEM) of the MPO units/mg of tissue. *p < 0.05 represents statistical differences compared to MTX group (-). The number of animals in each group was at least six. Data were analyzed by using analysis of variance (ANOVA) and Bonferroni tests.

MPO activity was significantly increased (p < 0.05) by the treatment with MTX in the all three small intestinal segments (duodenum, jejunum and ileum) in comparison to the untreated control group (control). Both aminoguanidine (10 mg/Kg) and L-NAME (20 mg/Kg) significantly (p < 0.05) reduced MTX-induced increase in MPO activity in duodenum, jejunum and ileum (Figure 3).

A significant (p < 0.05) increase in the jejunal MPO activity was observed in wild-type mice subjected to intestinal mucositis due to MTX treatment (iNOS^+/+^/MTX) in contrast to the control group, which received saline (iNOS^+/+^/control). MTX did not cause any inflammatory activity in the jejunum from inducible nitric oxide synthase (iNOS^-/-^) knock-out mice. The MPO activity was similar to the iNOS^-/-^/control, as well as to the wild-type iNOS^+/+^/control (p > 0.05) (Table 3).

**Table 3:** Myeloperoxidase (MPO) activity in the jejunum segment from inducible nitric oxide synthase knock-out mice (iNOS^-/-^) and C57BL/6 wild-type animals (iNOS^+/+^) submitted to intestinal mucositis

	iNOS^+/+^/control	iNOS^+/+^/MTX	iNOS^-/-^control	iNOS^-/-^/MTX
**Mean ± Std. error**	0.056 ± 0.01	8.45 ± 0.60 *	0.035 ± 0.02	0.056 ± 0.001

Intestinal mucositis was induced by subcutaneous administration of MTX. After sacrifice, on the 5^th ^day, a sample of the jejunum segment was harvested for MPO activity assay. *p < 0.05 represents statistical differences compared to iNOS^+/+^/control group. The number of animals in each group was at least four. Data were analyzed by using analysis of variance (ANOVA) and Bonferroni tests.

### 3.4. Cell proliferation and cell death

**Figure 4:**
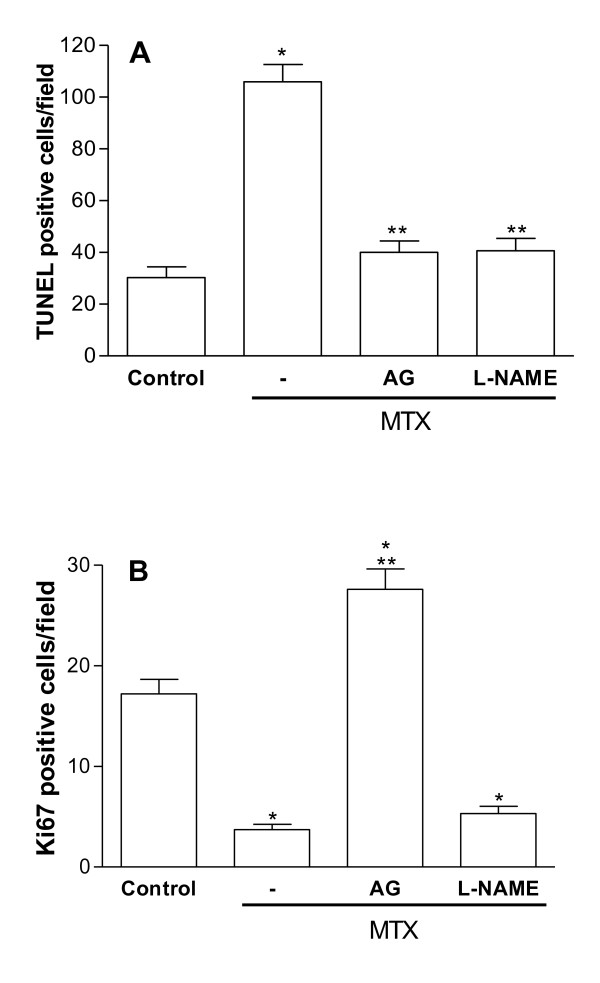
**Effect of aminoguanidine and L-NAME on cell death and proliferation in the jejunum segments**. An increased number of TUNEL positive cells is observed in jejunum of rats submitted to MTX-induced intestinal mucositis when compared to the jejunum of a normal control rat. The treatment with aminoguanidine or L-NAME significantly reduced the number of TUNEL positive cells in the lamina propria **(A)**. The treatment with MTX result in a significant decrease in the number of Ki67 positive cells compared to control animals. Aminoguanidine administration, but not L-NAME, result in a significant increase in the number of Ki67 positive cells compared to the MTX group (B). Bars represent the mean value ± standard error of the mean (SEM) of the tunel positive cells (A) and Ki67 positive cells (B). The TUNEL and Ki67 positive cells were counted (10 fields per slide; x1000) in order to perform a statistical comparison. *p < 0.05 represents statistical differences compared to control group. **p < 0.05 represents statistical differences compared to MTX group (-). Data were analyzed by using analysis of variance (ANOVA) and Bonferroni tests.

MTX-challenged jejunum showed a significant increase (p < 0.05) of TUNEL positive cells in the lamina propria, when compared to untreated control. Either aminoguanidine or L-NAME treatment substantially (p < 0.05) reduced the number of TUNEL positive cells in the lamina propria. When TdT enzyme was replaced by the reaction buffer, TUNEL positive cells were no longer detected (Figure 4A).

Figure 4B illustrates jejunum crypt cell proliferation (by Ki67 expression) in the four experimental groups. Treatment with MTX significantly (p < 0.05) reduced the number of Ki67 positive cells compared to control animals. This MTX effect was reversed by aminoguanidine but not by L-NAME treatment.

### 3.5. Immunohistochemical reaction for iNOS and nitrotyrosine

**Figure 5:**
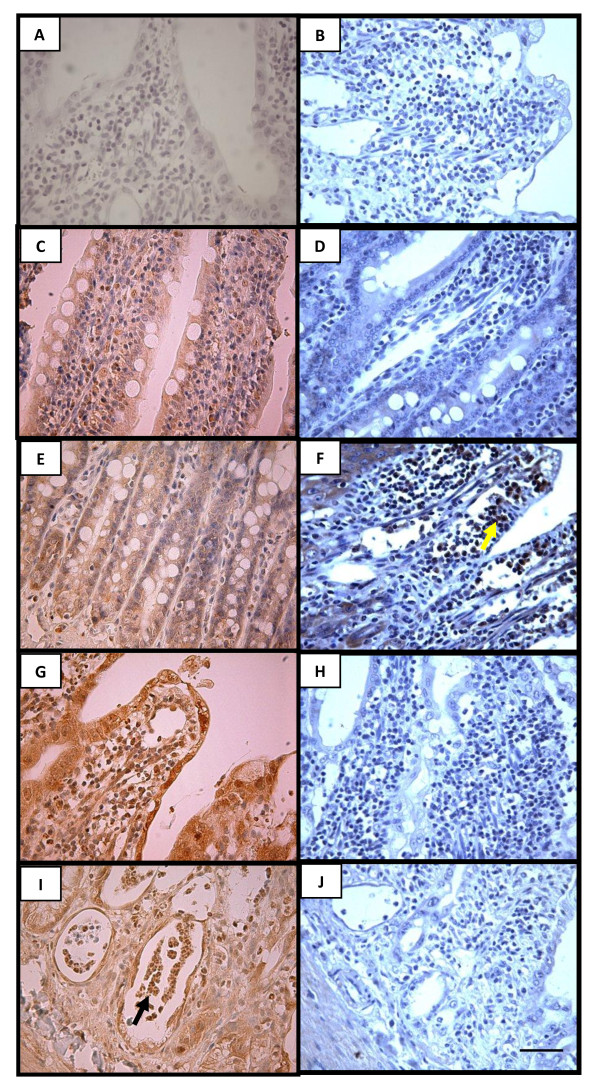
**Representative examples of iNOS (left) and nitrotyrosine (right) immunohistochemistry of rat jejunum**. The jejunum tissue of rats which received subcutaneous MTX presented intense immunostaining for iNOS, both in epithelial cells recovering the villi as well as in the lamina propria cells (G) and in the neutrophils (I; arrow) and other inflammatory cells surrounding and inside the necrotic crypts (I), when compared to the weak immunostaining in the jejunum villous and crypt region of a normal control rat (C and E). Negative control represents a sample of the jejunum where the antibody for iNOS was replaced by 5% PBS/BSA and no immunostaining was detected (A). The jejunum tissue of rats which received subcutaneous MTX presented intense immunostaining for nitrotyrosine in the lamina propria cells (F; yellow arrow), when compared to the weak immunostaining in the jejunum lamina propria and crypt region of a normal control rat (D). The treatment with aminoguanidine (H) or L-NAME (J) considerably reduced the immunostaining for nitrotyrosine. Negative control represents a sample of the jejunum where the antibody nitrotyrosine was replaced by 5% PBS/BSA and no immunostaining was detected (B). Magnification 400x. Scale bar lengths 10 μm. The yellow arrow indicate nitrotyrosine immunostained cells.

MTX-treated rats presented intense iNOS immunostaining in the jejunum enterocytes, lamina propria cells (Figure 5G), and neutrophils (Figure 5I; black arrow) and other inflammatory cells surrounding and within necrotic crypts (Figure 5I), when compared to the weak immunostaining of villus and crypt regions from unchallenged rats (Figure 5C and 5E). When the iNOS antibody was replaced by 5% PBS/BSA no immunostaining was detected (Figure 5A).

Rats receiving subcutaneous MTX presented intense immuno-labeling in the jejunum for nitrotyrosine both in enterocytes and lamina propria cells (Figure 5F), in contrast to the weak immunostaining seen in the unchallenged jejuni (Figure 5D). Either aminoguanidine (Figure 5H) or L-NAME (Figure 5J) treatment considerably reduced the immunostaining for nitrotyrosine. When nitrotyrosine antibody was replaced by 5% PBS/BSA no immunostaining was detected (Figure 5B).

## 4. Discussion

This study demonstrates that treatment with aminoguanidine and L-NAME, both NOS inhibitors, significantly prevented MTX-induced intestinal damage, such as villous atrophy, crypt necrosis and neutrophil infiltration, which is in accordance with a recent study by Kolli et al that suggested that nitrosative stress may play a role in MTX-induced mucositis [18]. The participation of NO in our model is supported by the increased iNOS expression in the intestinal tissue following MTX treatment. Furthermore, no significant inflammatory activity was found in jejunum of iNOS^-/- ^animals, as demonstrated by MPO assay.

Intestinal mucositis is a dose limiting side-effect of cancer chemotherapy, which leads to decreased absorption of nutrients, increased epithelial permeability, recurrent diarrhea, and weight loss [1]. MTX is a well-known cause of intestinal mucositis, which impairs rapidly dividing cells, such as epithelial stem cells within intestinal crypts, thereby causing diminished enterocyte replacement. Our group has previously demonstrated that MTX administration in rats causes villous atrophy with consequent reduction of the overall mucosal absorptive surface area [5]. It is well established that pro-inflammatory cytokines, such as interleukin-1 (IL-1), tumor necrosis alpha (TNF-α) and interleukin-6 (IL-6) are potent inducers of iNOS in a wide variety of cells types, with consequent production of NO [26,27]. Although the participation of pro-inflammatory cytokines in the intestinal mucositis has been shown [28,29], the role of nitric oxide is not fully understood.

It has been demonstrated that nitric oxide is an important mediator of 5-FU-induced oral mucositis [15], suggesting that chemotherapy-induced nitric oxide synthase (iNOS) activation may play a critical role in mucosal injury. In accordance, the present study demonstrates increased iNOS expression in jejunal samples as seen by immunohistochemistry, following treatment with MTX. Several studies have shown that sustained release of NO, as a result of iNOS upregulation, can lead to cellular damage and gut barrier failure [30] as reported in the experimental 2,4,6-trinitrobenzenesulfonic acid (TNBS)-induced colitis model in rats [31] and in guinea pig ileitis [32].

The iNOS immunostaining was particularly observed in neutrophils inside and surrounding the necrotic crypt, suggesting that NO produced by these cells is involved in the mucosa damage. The role of neutrophils on the MTX-induced intestinal mucositis was confirmed by increased MPO activity in all intestinal segments. We also observed that L-NAME and aminoguanidine protective effects were associated with reduced neutrophil infiltration (as seen as histopathology and MPO activity), suggesting that NOS inhibition might lead to poor neutrophil infiltration. A recent study by our group demonstrated that aminoguanidine and 1400 W reduced neutrophil infiltration in 5-FU-induced oral mucositis [15]. Indeed, the role of NO on neutrophil migration is still controversial, NO downregulates the expression of adhesion molecules in the vascular endothelium, therefore decreasing neutrophil trafficking into inflamed tissues [33,34]. On the other hand, in the rat skin, L-NAME can inhibit the edema formation induced by carrageenin, an inflammatory agent that promotes increased vascular permeability and massive leukocyte emigration, suggesting a pro-inflammatory role for NO in this model [35]. Moreover, during inflammatory reactions when large amounts of NO and superoxide are formed, the combination of both leads to the formation of reactive nitrogen species, such as the peroxynitrite [36]. This toxic compound has the ability to initiate lipid peroxidation, sulfhydryls oxidation, and readily nitrates phenolic compounds such as tyrosine residues on proteins [17,36-39], resulting in augmented inflammation and tissue injury. In this regard, we detected a significant increase of nitrotyrosine immunostaining in the intestinal segments of rats on the fifth day of the MTX-induced intestinal mucositis, reinforcing the role of NO via peroxynitrite on intestinal mucositis. Thus, the decrease in the neutrophil migration observed when aminoguanidine and L-NAME were administered to MTX-treated rats is probably related to suppression of peroxynitrite formation. Sustained iNOS upregulation, leading to increased NO levels, via peroxynitrite, has been shown to play a major role in the initiation of the intestinal mucosal injury in stressful conditions, such as endotoxemia, hemorrhagic shock, or necrotizing enterocolitis, causing enterocyte apoptosis and disruption of the intestinal barrier and increased bacterial translocation [40]. In the present study we demonstrate the role of NO on the peak of inflammation and tissue damage (5th after MTX-injection), which likely corresponds to the signal amplification/ulcerative phase of mucositis.

It is well known that high concentration of NO induces apoptosis in various cells including intestinal epithelial cells [40-43]. Accordingly, we found a significant increase in TUNEL-positive cells in the jejunum of rats following treatment with MTX compared to untreated controls. Both aminoguanidine and L-NAME were able to prevent cell death, as detected by the TUNEL assay. Additionally, we demonstrated that MTX significantly decreased the number of Ki67 positive cells indicating a reduction of proliferation. The treatment with AG, the more selective iNOS inhibitor, prevented the inhibitory effect of MTX on proliferation, suggesting that high levels of NO produced by iNOS may contribute to anti-proliferative action of MTX. Accordingly, the literature has shown a proliferative effect of AG in T-84 cell [44]. It has been reported that NO, via peroxynitrate, alters proliferative signaling mediated by protein tyrosine kinase, decreasing enterocyte proliferation which, in combination with NO-induced cell death, may contribute to gut barrier disruption [45]. As expected, L-NAME did not prevent the inhibitory effect of MTX on cell proliferation, since L-NAME itself may decrease cell proliferation in the intestinal epithelium [46].

## Conclusion

We have demonstrated that treatment with MTX induced intestinal epithelial damage in wild type rats and did not show a significant effect on intestinal inflammation in iNOS^-/- ^mice. The intestinal damage was prevented by NOS inhibitors L-NAME and AG. These findings suggest a critical role of NO, via the inducible iNOS, in MTX-induced intestinal mucositis.

## Authors' contributions

RL: Participated in the *in vivo *experiments, performed data analysis and drafted the manuscript. CV: Participated in the *in vivo *experiments, data storage, analysis and helped drafting the manuscript. JG: Participated in the *in vivo *experiments and data analysis. JE: Participated in the *in vitro *experiments and data analysis. CA: Participated in the study design and writing the paper. RB: Participated in statistical analysis and drafting and reviewing of the manuscript. GB and BO: Participated in the *in vivo *experiments and critical reviewing. GB and RR: Participated in the coordination, conception, experiments design and critical reviewing and writing. All authors read and approved the final manuscript.

## Acknowledgements

The authors thank Maria Silvandira França Pinheiro, Department of Physiology and Pharmacology, and José Ivan Rodrigues de Sousa, Department of Morphology, Faculty of Medicine, Federal University of Ceará, Brazil, for technical assistance. This work was supported by the Brazilian Agency for Scientific and Technological Development (CNPq) and Fundação Cearense de Apoio ao Desenvolvimento Científico e Tecnológico (FUNCAP).

## Disclosure Statement

The authors certify that there is no actual or potential conflict of interest in relation to this article.

## Pre-publication history

The pre-publication history for this paper can be accessed here:

http://www.biomedcentral.com/1471-230X/11/90/prepub
